# Role of Glucokinase in the Subcellular Localization of Glucokinase Regulatory Protein

**DOI:** 10.3390/ijms16047377

**Published:** 2015-04-02

**Authors:** Ling Jin, Tingting Guo, Zhixin Li, Zhen Lei, Hui Li, Yiqing Mao, Xi Wang, Na Zhou, Yizhuang Zhang, Ruobi Hu, Xuehui Zhang, Gang Niu, David M. Irwin, Huanran Tan

**Affiliations:** 1 Department of Pharmacology, Peking University, Health Science Center, Beijing 100191, China; E-Mails: jinling@bjmu.edu.cn (L.J.); ttguo@bjmu.edu.cn (T.G.); lihui@bjmu.edu.cn (H.L.); maoyiqing@bjmu.edu.cn (Y.M.); xixi1125@bjmu.edu.cn (X.W.); zhouna@bjmu.edu.cn (N.Z.); onepiecezyz1989@163.com (Y.Z.); huruobi@bjmu.edu.cn (R.H.); xuehuizhang@bjmu.edu.cn (X.Z.); 2 Department of Integrated Traditional Chinese and Western Medicine, Peking University, Health Science Center, Beijing 100191, China; E-Mail: leezhixin@tom.com; 3 Department of Pharmacology, Ningxia Medical University, Yinchuan 750004, China; E-Mail: lei1153@163.com; 4 Beijing N&N Genetech Company, Beijing 100082, China; 5 Department of Laboratory Medicine and Pathobiology, University of Toronto, Toronto, ON M5S 1A8, Canada

**Keywords:** protein–protein interaction, glucokinase regulatory protein, glucokinase, sub-cellular localization, glucose metabolism

## Abstract

Glucokinase (GCK) is the rate-limiting enzyme of liver glucose metabolism. Through protein-protein interactions, glucokinase regulatory protein (GCKR) post-transcriptionally regulates GCK function in the liver, and causes its nuclear localization. However the role of GCK in regulating GCKR localization is unknown. In the present study, using *in vitro* and *in vivo* models, we examined the levels of GCK and GCKR, and their subcellular localization. We found that total cellular levels of GCKR did not vary in the *in vivo* models, but its subcellular localization did. In animals with normal levels of GCK, GCKR is mainly localized to the nuclei of hepatocytes. In seven-day old rats and liver-specific *Gck* gene knockout mice (animals that lack or have reduced levels of GCK protein), GCKR was found primarily in the cytoplasm. The interaction of GCK with GCKR was further examined using *in vitro* models where we varied the levels of GCK and GCKR. Varying the level of GCK protein had no effect on total cellular GCKR protein levels. Taken together, our results indicate that GCK is important for the localization of GCKR to the nucleus and raises the possibility that GCKR may have functions in addition to those regulating GCK activity in the cytoplasm.

## 1. Introduction

Glucokinase regulatory protein (GCKR) is a 68 kDa protein that is mainly expressed in the liver [1] and interacts with glucokinase (GCK), the key regulator of glucose metabolism [2]. GCKR interacts with GCK through a binding site that has two affinities, low and high, to regulate the enzymatic activity of GCK [3], with these interactions leading to the inhibition of enzymatic activity and nuclear localization [4,5,6,7,8]. Fructose regulates the binding of GCKR and GCK, with fructose-6-phosphate increasing, and fructose-1-phosphate decreasing, the binding affinity at a single site [9]. In addition to causing a nuclear localization, GCKR stabilizes GCK and prevents its degradation [4,5,6]. Mice deficient in GCKR have normal or even increased levels of *Gck* mRNA but have decreased amounts of GCK protein and enzymatic activity [4,5]. Fasting *Gckr*^−/−^ mice treated with insulin show an increase in their *Gck* mRNA levels, but little effect on GCK protein levels [4]. Similarly, HepG2 hepatoma cells transduced with adenoviral vectors encoding both GCK and GCKR yielded increased GCK activity and protein levels compared to cells transduced by GCK alone [6]. GCK exists exclusively in the cytoplasm of *Gckr* knockout mice [4,5]. Similarly, when GCK is transfected alone into cells, GCK is mainly distributed in the cytoplasm; however, when both GCK and GCKR are co-transfected into cells then most of the GCK is found in the nucleus [7,8]. These studies indicate that the level of GCRK is important for regulating the level and subcellular localization of GCK, however, most of these cell line studies did not use hepatic cells, but rather heterologous systems, thus should be confirmed in a more normal cellular environment. Fewer studies have examined the role of GCK in regulating GCKR protein levels or localization. Previous studies have reported that, while hexokinase levels remain constant, GCK begins to appear in the liver of the rat at approximately 16 days after birth, and reaches adult levels about 10–12 days later [10]. Expression of *Gckr*, however, has been reported to start four days before birth [11]. A heterozygous liver-specific *Gck* gene (*Gck*^w/−^) knockout mouse has been generated [12] that results in a liver-specific decrease in GCK protein and enzymatic activity, but does not affect pancreatic GCK function at a young age [11,12,13]. We have used these animal models, along with hepatic cell line models, to examine the role of GCK in regulating the levels and sub-cellular localization of GCKR.

## 2. Results and Discussion

### 2.1. Sub-Cellular Localization of Glucokinase Regulatory Protein (GCKR) in Hepatic Cell Lines

**Figure 1:**
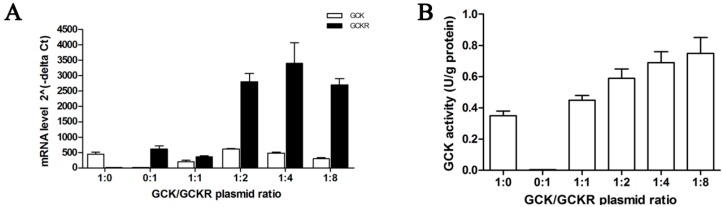

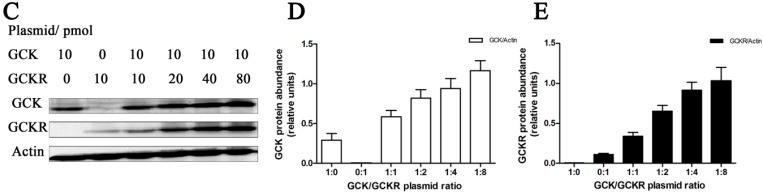
Expression of glucokinase (GCK) and glucokinase regulatory protein (GCKR) in co-transfected liver L-02 cells. GCK and GCKR expressing plasmids were co-transfected into L-02 cells at a range of plasmid ratios (molar) from 1:1 to 1:8. Cells transfected with the GCK expressing, GCKR expressing, and GCKR backbone plasmids were used as controls. (**A**) Quantitative real-time RT-PCR assessment of the mRNA levels of *Gck* (open bars) and *Gckr* (solid bars) at different plasmid ratios; (**B**) GCK enzymatic activity at different plasmid ratios; (**C**) Representative Western blots for GCK and GCKR, with β-actin used as a loading control, at different plasmid ratios; and (**D**,**E**) Quantification of the immunoblots for GCK (**D**) and GCKR (**E**), with relative units for GCK (**D**) or GCKR (**E**) abundance being the abundance of GCK or GCKR normalized to the β-actin level for that sample. Data are presented as means ± S.D. (Standard Deviation) (*n* = 4).

Previous studies investigating GCKR function and sub-cellular localization typically used liver tissue or hepatocytes from adult rats [14,15] or non-liver cell lines [16,17]. Here, we used the normal hepatic cell line L-02 [18] and the cancer derived human hepatoma cell line HepG2 [19]. Hepatic cell lines endogenously express little if any GCK or GCKR [6], thus we transfected plasmids encoding human GCK and/or GCKR coding sequences into these cells alone or at a range of plasmid ratios. Increases in the amount of GCKR plasmid led to increased levels of *Gckr* mRNA and protein up to the 1:4 ratio (Figure 1A,E). While *Gck* mRNA levels remained similar in these transfections, GCK enzymatic activity and protein levels increased with increasing GCKR plasmid ratios (Figure 1A,B,D). Increased GCK activity and protein level is in accord with previous studies showing that GCKR stabilized GCK protein levels by preventing its degradation [6]. To determine whether the GCK and GCKR expression observed in the transfected cells was due to transcription from the plasmids or endogenous genes, RT-PCR that could distinguish plasmid and endogenous derived mRNAs was conducted. All GCK and GCKR transcripts detected by RT-PCR in the transfected cells were derived from the plasmids (Figure S1). These data also indicate that we successfully constructed a GCK and GCKR expressing liver cell line. A GCK:GCKR plasmid ratio of 1:4 was used for the remaining experiments, as this ratio appears to mimic the endogenous hepatocyte *Gck*/*Gckr* mRNA ratio [20].

**Figure 2:**
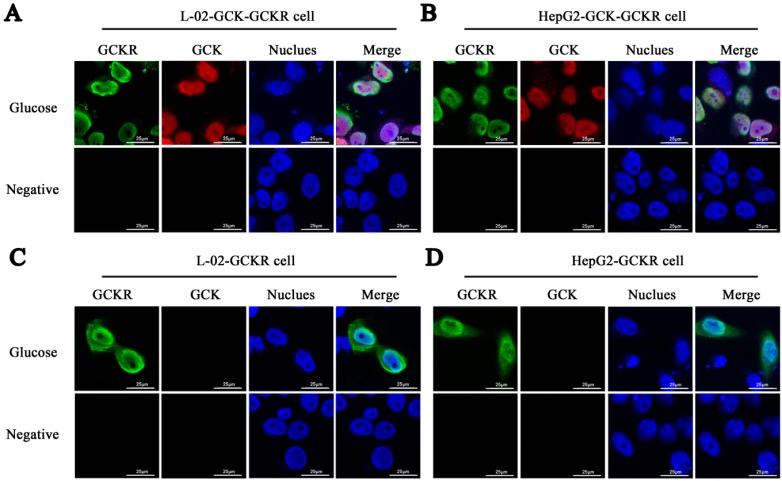

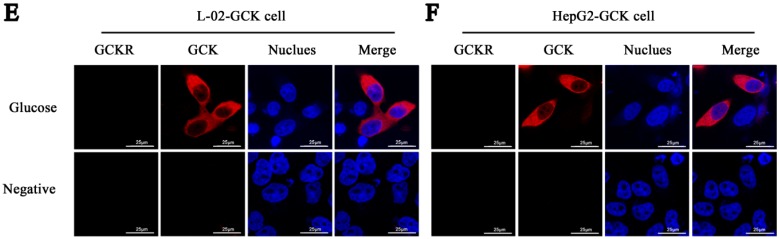
Sub-cellular localization of GCK and GCKR in liver L-02 and HepG2 cells in glucose containing media. GCK and/or GCKR expressing plasmids were transfected into liver cell lines. Forty-eight hours after transfection, culture medium was changed to low glucose (5.5 mM glucose). Confocal immunofluoresence micrographs are shown for cells after culturing in low glucose for 2 h. (**A**) L-02 cells co-transfected with GCK and GCKR plasmids; (**B**) HepG2 cells co-transfected with GCK and GCKR plasmids; (**C**) L-02 cells transfected with GCKR plasmids; (**D**) HepG2 cells transfected with GCKR plasmids; (**E**) L-02 cells transfected with GCK plasmids; and (**F**) HepG2 cells transfected with GCK plasmids. GCKR (revealed in green) and GCK (revealed in red) were detected using antibodies, and the nuclei were counterstained with Hoechst (stained in blue). The overlap of the three channels is shown as Merge. White color in the merged panels in (**A**) and (**B**) denotes co-localization of GCKR and GCK in the nucleus. Cells incubated with only secondary antibodies were used as negative controls. Images are representative of four experiments with similar results. Scale bar = 25 μm.

The localization of GCKR in co-transfected hepatic cells lines was examined by immunostaining, simultaneously, with Dylight 594 for GCK and Dylight 488 for GCKR. GCKR stayed in the nucleus of the transfected cells when they were cultured in glucose medium, as did GCK (Figure 2A,B). The intracellular location of GCK, however, in the co-transfected cells was not modulated by the concentration of glucose, in contrast to *in vivo* results [14,15,21]. This difference may be due to many factors such as differences in energy status of the primary cells *versus* cell lines or differences in the expression of glucose transporters, other hexokinases, and other genes involved in the glycolytic flux [8,22,23,24]. Previous reports using GCK and GCKR expressing plasmids in the non-liver HeLa cells showed similar results, with both proteins remaining in the nucleus even when cells were cultured in 25 mM glucose [17,25]. In cells transfected with only the GCKR or GCK plasmid, GCKR was found in both the nucleus and the cytoplasm (Figure 2C,D), while GCK remained in the cytoplasm (Figure 2E,F) in both low and high glucose media.

### 2.2. Direct Interaction of GCKR and Glucokinase (GCK) in the Nuclei of Co-Transfected Hepatic Cell Lines

**Figure 3:**
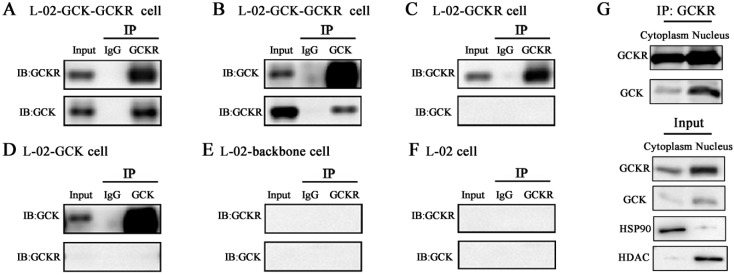
Interaction of GCK and GCKR in whole cell lysates, and nuclear fractions, of co-transfected L-02 cells. L-02 cells were transfected with GCKR and/or GCK expressing plasmids. Total protein cellular lysates were immunoprecipitated with anti-GCKR (**A**,**C**,**E**,**F**) or anti-GCK (**B**,**D**) followed by immunoblotting with anti-GCK and anti-GCKR. Cytoplasmic and nuclear protein cellular lysates were immunoprecipitated with anti-GCKR followed by immunoblotting with anti-GCK and anti-GCKR (**A**,**G**). Immunoprecipitation of GCKR in the co-transfected L-02 cells; (**B**) Immunoprecipitaion of GCK in the co-transfected L-02 cells; (**C**) Immunoprecipitation of GCKR in GCKR plasmid transfected cells; (**D**) Immunoprecipitation of GCK in GCK plasmid transfected cells; (**E**,**F**) No immunoprecipitation of GCK and GCKR in cells transfected by the backbone vector (**E**) and in untransfected L-02 cells (**F**,**G**). Cytoplasmic and nuclear immunoprecipitation of GCKR in the co-transfected L-02 cells. Hsp90 and histone deacetylase (HDAC) were used as cytoplasmic and nuclear fraction markers, respectively. Input represents cell lysates before co-IP. Data are representative of four experiments with similar results.

Co-immunoprecipitation was performed to determine whether there was a direct interaction between GCK and GCKR in the co-transfected hepatic cell lines. As shown in Figure 3, reciprocal immunoprecipitation of GCK and GCKR in the L-02 cells shows that GCK and GCKR interact with each other (Figure 3A,B). No additional interacting proteins were detected in the GCK or GCKR transfected cells (Figure 3C,D). GCKR and GCK protein bands were not detected in control or untransfected L-02 cells (Figure 3E,F). These data demonstrate that GCK binds to GCKR in L-02 cells. Cytoplasmic and nuclear proteins were separated from the co-transfected hepatic cells for co-immunoprecipitation. Larger amounts of GCK protein were detected in the nuclear fraction than the cytosolic fractions when pulled down by GCKR (Figure 3G), indicating that the interaction of these two proteins results in largely a nuclear localization.

### 2.3. Sub-Cellular Localization of GCKR in Seven-Day Old Adult Rats

**Figure 4:**
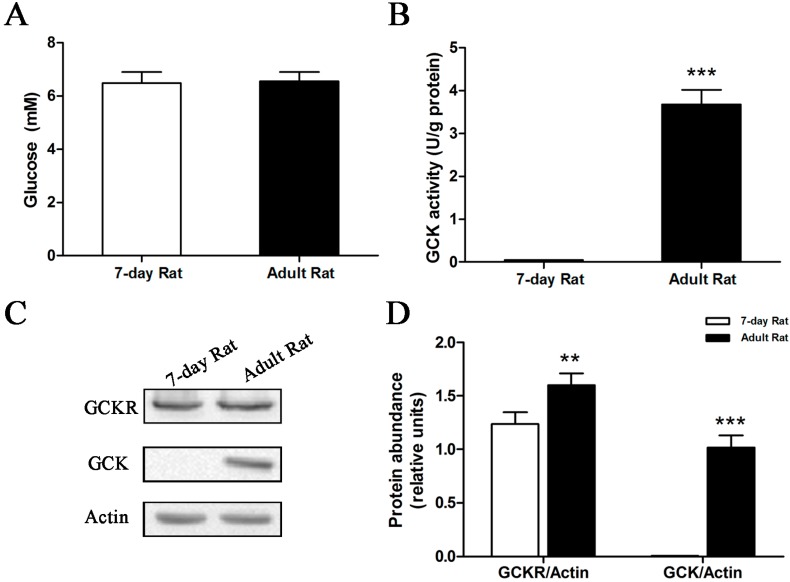
Glucokinase activity and protein levels in seven-day old rats. Rats, seven-day old and adults, had free access to food and water. (**A**) Serum glucose concentrations; (**B**) GCK enzymatic activity from liver extracts; (**C**) Representative Western blot of GCK and GCKR protein levels, with β-actin used as a loading control; and (**D**) Quantification of the GCK and GCKR immunoblots, for seven-day old and adult rats. Relative units for GCK and GCKR protein abundance in (**D**) are the abundance of GCK or GCKR normalized to the β-actin levels of that sample. ** *p* < 0.01, *** *p* < 0.001 *vs.* seven-day old rat. Data are presented as means ± S.D. (*n* = 4).

Previous studies reported that, while hexokinase levels remain constant, GCK begins to appear in the liver of the rat at approximately 16 days after birth, and reaches adult levels about 10–12 days later [10]. Expression of *Gckr*, however, has been reported to start four days before birth [11]. Our result confirmed this (Figure S2). Western blots show that GCK protein is absent during the first two weeks of extrauterine life, and that the levels gradually increase at older ages. A statistically significant difference (*p* < 0.001) in GCK protein levels is seen between the seven-day old and adult rats. Blood glucose levels were almost the same between seven-day old and adult rats in the *ad libitum* fed state (Figure 4A); However, clear differences in GCK activity and protein levels were seen between these groups. Both GCK activity (Figure 4B) and protein (Figure 4C,D) are absent in seven-day old rats.

**Figure 5:**
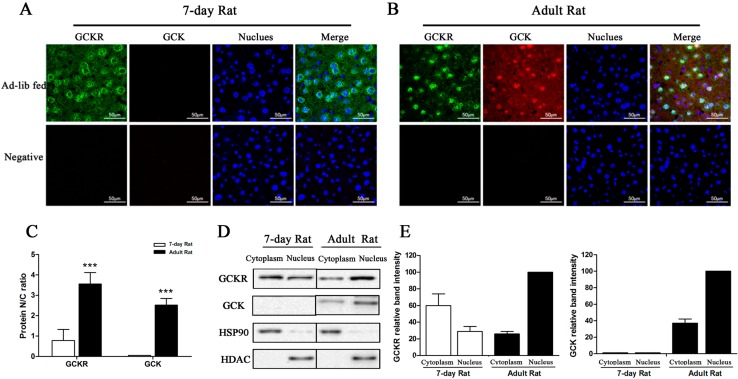
Sub-cellular localization of GCKR in seven-day old rats. (**A**,**B**) Confocal immunofluoresence micrographs with antibodies against GCKR (revealed in green), GCK (revealed in red) with nuclei counterstained with Hoechst (stained in blue) in seven-day old (**A**) and adult (**B**) rats. Overlap of the three channels is shown in Merge. White color in the merged panels denotes co-localization of GCKR and GCK in the nucleus. Slides incubated with only the secondary antibodies were used as negative controls. Images are representative of four experiments with similar results. Scale bar = 50 μm; (**C**) Nuclear/cytoplasmic ratio (N/C ratio) for GCKR and GCK derived from immunoflouresence. Data are presented as means ± S.D. from four individual hepatocyte preparations with 30 cells in total. *** *p* < 0.001 *vs.* 7-day old rat. (**D**) Representative Western blots for GCKR and GCK protein from the cytosolic (**C**) and nuclear (N) fractions. Abundance of GCK and GCKR in the cytosolic and nuclear fractions was normalized for comparable amounts of protein. Hsp90 and HDAC are markers for the cytoplasmic and nuclear fractions, respectively; (**E**) Densitometry analysis of the immunoblots in (**D**). Intensity of the protein band in the nuclear fraction of the adult rat is set as 100. Data are representative of four experiments with similar results.

The intracellular localization of GCKR was examined using confocal immunomicroscopy. As shown in Figure 5A,B, GCKR distribution in the liver cells differed markedly between the seven-day old and adult rats. Both cytosolic and nuclear fluorescence signals were observed in seven-day old rats (Figure 5A), while an intense fluorescent signal was detected only in the nuclei from adult rats (Figure 5B). The GCKR nuclear/cytosolic (N/C) ratio for seven-day old rats was much lower than for adult rats (Figure 5C). GCK was not visible in hepatocytes from seven-day old rats, but was found in the nucleus and cytoplasm of the adult (Figure 5A,B). Intracellular locations of GCK and GCKR were confirmed using nuclear and cytosolic fractions of hepatocyte lysates probed with anti-GCK or anti-GCKR antibodies. Western blots showed that GCKR was present in both the cytosol and nucleus in seven-day old rats, while in adults the majority of the GCKR was located in the nucleus, as seen by immunomicroscopy (Figure 5D,E).

### 2.4. Sub-Cellular Localization of GCKR in Liver-Specific Gck Gene Knockout (Gck^w/−^) Mice

Heterozygous liver-specific *Gck*^w/−^ mice, in a C57BL/6J background, were previously constructed by our lab [12]. Fasting glucose levels in these *Gck*^w/−^ mice were mildly, but significantly, increased compared with wild-type mice (*p* < 0.05) (Figure S3A). No significant change in body weight was noted between the *Gck*^w/−^ and wild-type mice (Figure S3B). Intraperitoneal glucose tolerance test (IPGTT) and total area under the curve (AUC) were used to evaluate their glucose tolerance. IPGTT displayed a diabetic curve in the *Gck*^w/−^ mice, where the blood glucose levels at 120 min were significantly higher than those at 0 min (*p* < 0.01) and also higher than that seen in age matched *Gck*^w/w^ mice at 120 min (*p* < 0.001) (Figure S3C). The AUC_glucose_ for the *Gck*^w/−^ mice was much larger than in *Gck*^w/w^ mice (*p* < 0.01) (Figure S3D). These data demonstrate the diabetic characteristics of the liver-specific *Gck* gene knockout mice.

**Figure 6:**
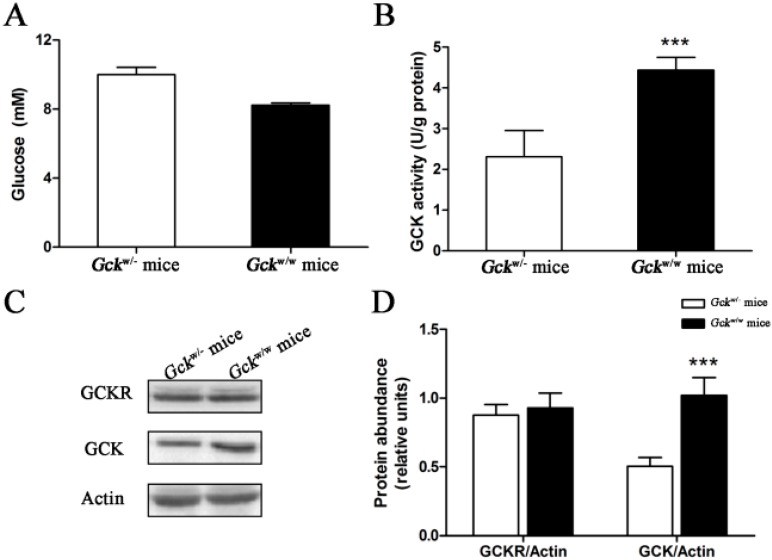
Enzymatic and protein levels for glucokinase in the liver-specific *Gck* gene knockout (*Gck*^w/−^) mice. *Ad libitum* fed mice had free access to food and water. (**A**) Serum glucose concentrations; (**B**) GCK activity in the liver; (**C**) Representative Western blots for GCK and GCKR, with β-actin as a loading control; and (**D**) quantification of the immunoblots of glucokinase and GCKR are shown for *Gck*^w/−^ and *Gck*^w/w^ mice in the *ad libitum* fed state Relative units for GCK and GCKR protein abundance in (**D**) are the abundance of GCK or GCKR normalized to the β-actin level of that sample. *** *p* < 0.001 *vs.* *Gck*^w/−^ mice. Data are presented as means ± S.D. (*n* = 4).

*Gck*^w/−^ mice, where one allele of the *Gck* gene in the liver is disrupted, demonstrate a slightly higher blood glucose level in *ad libitum* fed state (Figure 6A). As expected, GCK enzymatic activity of *Gck*^w/−^ mice is about 50% of the level of *Gck*^w/w^ mice (*p* < 0.001) (Figure 6B). Preliminary real-time quantitative PCR experiments measuring *Gck* and *Gckr* mRNA indicated that the *Gck* mRNA levels in *Gck*^w/−^ mice were about half those of *Gck*^w/w^ mice, but no difference in *Gckr* mRNA levels were detected (Figure S4). Western blots displayed a markedly reduced amount of GCK protein in knockout mice, of about half the level of wild-type animals (*p* < 0.001), however, no difference in GCKR protein levels was seen (Figure 6C,D). The significant, and parallel, 50% reductions in the levels of *Gck* mRNA and protein in the liver-specific *Gck* knockout mice suggest that post-translational events are not responsible for the decrease in GCK activity.

**Figure 7:**
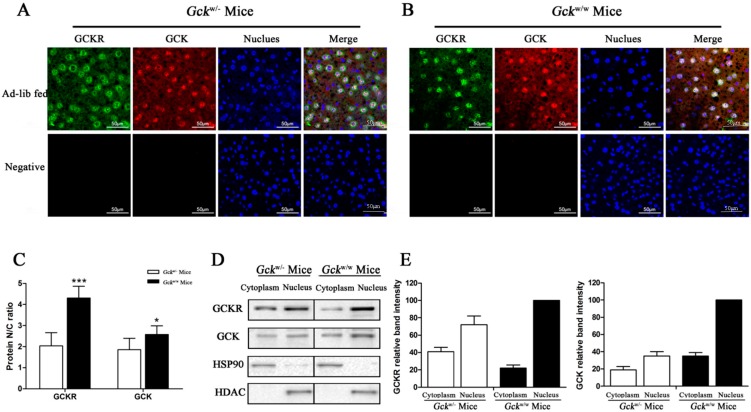
Sub-cellular localization of GCKR in the liver-specific *Gck* gene knockout (*Gck*^w/−^) mice. (**A**,**B**) Confocal immunofluoresence micrographs with antibodies against GCKR (revealed in green), GCK (revealed in red) with the nuclei counterstained with Hoechst (stained in blue) in (*Gck*^w/−^) (**A**) and wild-type (*Gck*^w/w^) (**B**) mice. Overlap of the three channels is shown in Merge. White color in the merged panels denotes co-localization of GCKR and GCK in the nucleus. Slides incubated with only the secondary antibodies were used as negative controls. Images are representative of four experiments with similar results. Scale bar = 50 μm; (**C**) Nuclear/cytoplasmic ratios (N/C ratio) for GCKR and GCK are based on the immunoflouresence. Data are presented as means ± S.D. from four individual hepatocyte preparations with 30 cells in total. * *p* < 0.05, *** *p* < 0.001 *vs.* *Gck*^w/−^ mice; (**D**) Representative Western blots for GCKR and GCK protein in the cytosolic (**C**) and nuclear (N) fractions. Cytosolic and nuclear fractions are normalized for comparable amounts of protein. Hsp90 and HDAC were used as cytoplasmic and nuclear fraction markers, respectively; (**E**) Densitometry analysis of the immunoblot is shown in (**D**). Relative band intensity of the proteins in the nuclear fraction of the *ad libitum* fed wild-type mice is set as 100. Data are representative of four experiments with similar results.

Since the amount of GCKR in liver cells was not affected by the amount of GCK (Figure 6C,D), we next examined the subcellular localization of both GCKR and GCK in the livers of *Gck*^w/−^ and *Gck*^w/w^ mice. Subcellular localization was visualized by immunofluorescence confocal microscopy. As expected, GCKR was mainly located in the nucleus of the *Gck*^w/w^ mice, (Figure 7B). In contrast, a larger portion of the GCKR was detected in the cytoplasm in *Gck*^w/−^ mice (Figure 7A). A significant decrease (*p* < 0.001) in the GCKR N/C ratio was observed in the knockout mice (Figure 7C), and since the total cellular levels of GCKR are equal in these two mice (Figure 6C,D), this indicates that a greater amount of GCRK accumulates in the cytoplasm of *Gck*^w/−^ mice. For GCK, *Gck*^w/−^ mice showed slightly higher amounts of GCK in the cytoplasm and a slightly lower GCK N/C ratio (*p* < 0.05) compared to wild-type mice (Figure 7A–C), suggesting that only a small amount of GCK moved to the cytoplasm. Nuclear and cytoplasmic fractions from liver cells were used to verify the localization of GCK and GCKR. Western blot analysis of the cell fractions revealed increased amounts of GCKR in the cytosol of *Gck*^w/−^ mice compared to wild-type mice (Figure 7D,E). The subcellular localization patterns in wild-type and knockout mice was similar to those observed by immunofluorescence.

## 3. Discussion

Using both *in vitro* (transfected hepatic cell lines) and *in vivo* (young and old rats and liver specific *Gck* knockout mice) that have differential expression of GCK, we were able to determine the effect of GCK on the subcellular localization of GCKR. In all models, decreased levels of GCK lead to increased GCKR localization to the cytoplasm of hepatic cells (Figure 5A,B and Figure 7A,B). These results are consistent with the model that GCKR and GCK are localized to the nucleus when they are in a complex, but that when they are not bound to each other however, they can move to the cytoplasm [4,8,14,15,21,26]. While small particles can pass through the nuclear pore complex by passive diffusion, larger particles (>40 kDa), such as GCK and GCKR, require active transport [27]. Export and import of protein to the nucleus are facilitated in part by nuclear export signal (NES) and nuclear localization signal (NLS) sequences [28,29]. GCK lacks a NLS but does contain a leucine-rich NES [8], which means that GCK can be exported from the nucleus when its entry needs assistance. GCKR has been proposed to be involved in GCK nuclear entry, using a piggy-back mechanism [8]. In response to high glucose levels (via fructose-1-phosphate), GCK dissociates from GCKR and can be exported from the nucleus using its own NES [8,15,16,21,26]. While GCK is exported in response to high glucose, the fate of GCKR is controversial. Some studies have concluded that GCKR is exported [4,14,21,26,30], while others reach an opposite conclusion [31,32]. As the turnover rate of GCKR is slower than that of GCK [1], and GCKR appears to assist in the transport of GCK into the nucleus, the possibility that a small amount of GCKR moves to cytoplasm from the nucleus cannot be ruled out. Our immunolocalization analyses and Western blot on the animal models are consistent with some GCKR moving from the nucleus or staying in the cytoplasm.

Reduced nuclear GCKR localization in the presence of reduced levels or the absence of GCK, may explain reduced nuclear localization of GCKR. However, other factors may also explain our results. Reduced GCKR nuclear localization in the seven-day old rats might be due to an incompletely developed nuclear shuttle mechanism, compared to adult rats. The liver-specific *Gck*^w/−^ mice show mild glucose elevation [12,13,33], thus diabetes might contribute to the cytoplasmic GCKR localization. This explanation would be consistent with the cytoplasmic localization of GCK and GCKR in the liver of the Zucker diabetic fatty rats, despite this animal having normal levels of GCK and GCKR, and serious hyperglycemic (~30 mM) blood glucose levels [34,35]. Intriguingly, in the human liver, *Gckr* mRNA expression is in molar excess when compared with *Gck* [20]; For the liver of adult fed rats, the GCKR level is threefold molar higher [36]. While GCKR is always in excess of GCK , the ratio for these two proteins when interacted is 1:1 [36]. In addition, *Gckr* is expressed before birth in the rat, but *Gck* is not expressed until day 16 after birth [30]. Further, our experiments showed that decreased levels of GCK lead to increased GCKR localization to the cytoplasm of hepatic cells (Figure 3A,B and Figure 4A,B). Taken together, these observations raise the possibility that GCKR may have functions other than being a regulator of GCK in the cytoplasm, especially in the first two weeks after birth in the rat.

## 4. Experimental Section

### 4.1. Cell Culture, Plasmids and Transfection

Human heptoma cell line HepG2 (ATCC, Manassas, VA, USA) and the normal human liver cell line L02 (Type Culture Collection of the Chinese Academy of Sciences, Shanghai, China) were grown in Dulbecco’s Modified Eagle Medium (Life technologies, New York, NY, USA) supplemented with 10% fetal bovine serum FBS. Plasmids encoding human glucokinase (pCMV6-XL5-GCK, sc305649), and human glucokinase regulatory protein (pCMV6-XL4-GCKR, sc119244) were purchased from OriGene (Rockville, MD, USA). Transfections were performed at 50%–70% cell confluence, following the instructions of the Xfect transfection reagent (Takara, Shiga, Japan). Forty-eight hours after transfection, cells were harvested for immunofluorescence and immunoprecipitation.

### 4.2. Animals

Adult rats (200–220 g), seven-day rats (18–21 g), 10-week liver-specific heterozygous *Gck*^w/−^ mice, and age-matched *Gck*^w/w^ mice were used, with free access to food and water. All animals were accommodated in standard conditions (temperature 21 ± 2 °C, 12:12 light-dark cycle, lights on at 8:00 am). Animal experiments were performed in accordance with the “Guidelines for Animal Experiment” and approved by the Animal Care Committee of the Peking University Health Science Center.

### 4.3. Blood Glucose and Glucokinase Activity

Blood glucose was measured from the tails of the mice using the Glucotrend 2 blood glucose monitor (Roche, Basel, Switzerland). Glucokinase activity was measured as previously described [5].

### 4.4. Immunofluorescence and Confocal Microscopy

Livers tissue and cells were fixed and blocked. Then, anti-GCK antibody (sc-7908, Santa Cruz, CA, USA) and anti-GCKR antibody (sc-6340, Santa Cruz) were mixed together to react with the antigens. The following day, DyLight 596-conjugated antibody (DkxRb-003-D594NHSX, ImmunoReagents, Raleigh, NC, USA) and DyLight 488-conjugated antibody (E032231, Earthox, San Fransisco, CA, USA) were mixed to incubate with the primary antibodies Hoechst 33258 (C1017, Beyotime, Shanghai, China) were applied to stain the nuclei. Confocal fluorescence images were captured with Leica TCS SP5 confocal microscope (Leica, Wetzlar, Germany). Immunofluorescence intensities were quantified by LAS AF Lite software (Leica), where equal areas of cytoplasm and nucleus were measured for each cell. The nuclear/cytoplasmic ratio (N/C ratio) was calculated by dividing the nuclear intensity to the cytoplasmic intensity.

### 4.5. Preparation of Total, Cytoplasmic, and Nuclear Protein Extracts

Total protein was homogenized in lysis buffer containing 50 mM Tris HCl (pH 8.0), 150 mM NaCl, 0.1% SDS, 1 mM EDTA, 0.5% Sodium deoxycholate, 100 mg/mL phenylmethanesulfonylfluoride (PMSF) and 1% NP-40. Nuclear and cytosolic fractions were obtained using the NE-PER Nuclear and Cytoplasmic Extraction Reagent kit (Thermo, San Jose, CA, USA). Protein concentrations in all samples were determined using the BCA method (Yuanpinghao, Tianjing, China).

### 4.6. Co-Immunoprecipitation

Pre-cleared lysates from total proteins and nuclear fractions were incubated overnight with anti-GCK antibody (sc-7908, Santa Cruz), anti-GCKR antibody (sc-6340, Santa Cruz) and isotype antibodies as a negative control. Protein G-agarose beads (Roche) were added with gentle rocking, and then washed three times with lysis buffer.

### 4.7. Western Blotting

Proteins were separated by SDS-PAGE and electrotransferred to PVDF membranes (Millipore, Bedford, MA, USA). Blots were visualized by chemiluminescence ChemiDoc XRS (Bio-Rad, Hercules, CA, USA), and the intensities of bands were measured by Quantity One v 4.6.2 software (Bio-Rad). Primary antibodies used in the study included anti-GCK antibody (sc-7908, sc-1979, Santa Cruz), anti-GCKR antibody (sc-6340, Santa Cruz), anti-β-actin antibody (#4967, CST), anti-HSP90 (BS1181, Bioworld, Louis Park, MN, USA), and anti-HDAC (BS1162, Bioworld).

### 4.8. Isolation of Total RNA, Quantitative RT-PCR and RT-PCR

Total RNA was isolated according to the instructions of the RNeasy Mini kit (Qiagen, Valencia, CA, USA). cDNA was synthesized using the Primescript 1st strand cDNA synthesis kit (Takara, Shiga, Japan). Quantitative detection of *Gck* and *Gckr* mRNA levels was performed using the Miniopticon Real-Time PCR Detection system (Bio-Rad) with iQ SYBR Green PCR SuperMix (Bio-Rad). Primers for real-time PCR are listed in Table 1. For qualitative detection of *Gck* and *Gckr* expression, two sets of primers were designed, with reverse primers specific to the CDS regions and forward primers specific to the 5' vector or endogenous 5' untranslated regions. Primers for RT-PCR are listed in Table 2.

**Table 1:** Primers for real-time RT-PCR analysis.

Gene	Primer
Human Glucokinase (h*Gck*)	F-GAATGACACGGTGGCCACGATG
	R-CACTCGGTATTGACGCACATGCG
Mouse Glucokinase (m*Gck*)	F-GAATCTTCTGTTCCACGGAG
	R-AGTGCTCAGGATGTTAAGGA
Human Glucokinase regulatory protein (h*Gckr*)	F-GGTGGAAGTGCCACCAAGATTCTGC
	R-GTCTGCCAGCCAACCAGGTACACG
Mouse Glucokinase regulatory protein (m*Gckr*)	F-AGGCATTTCCGTGGGACTCTC
	R-ACCGGATTGAAGCCAACCAG
Human Actin	F-TTCGAGGCTTTCAACACACCAG
	R-GGCATGAGGCAGGGCATAAC
Mouse Actin	F-CATCCGTAAAGACCTCTATGCCAAC
	R-ATGGAGCCACCGATCCACA

**Table 2:** Primers for RT-PCR analysis.

Gene	Primer
Glucokinase (*Gck*)	F-ATGTCACAAGGAGCCAGGCCCAGA
	R-ACTTCTGAGCCTTCTGGGGTGGA
Glucokinase vector	F-GCGTGTACGGTGGGAGGTCTATAT
	R-ACTTCTGAGCCTTCTGGGGTGGA
Glucokinase regulatory protein (*Gckr*)	F-GCAAGTGGGAGTTGTCTGGGTAC
	R-CAATCCCGTGCAAGGCACTATCT
Glucokinase regulatory protein vector	F-AGGCGTGTACGGTGGGAGGTCTAT
	R-GGCTCCTTCAGCACTTCCTGAACTT

### 4.9. Statistical Analysis

Results are shown as means ± standard deviation (S.D.). Statistical analysis was performed by *t* tests, one-way ANOVA followed by a Bonferroni’s test, and two-way ANOVA using a Bonferroni’s test. A probability of less than 5% was considered to be significant (*p* < 0.05).

## 5. Conclusions

Our results demonstrate that GCK is important for the localization of GCKR to the nucleus. It also raises the possibility that GCKR may have functions in addition to those regulating GCK activity in the cytoplasm.

## Supplementary Materials

Supplementary materials can be found at http://www.mdpi.com/1422-0067/16/04/7377/s1.
