# Supplementary material for: Role of Glucokinase in the Subcellular Localization of Glucokinase Regulatory Protein
Source: Int J Mol Sci. 2015 Apr 2;16(4):7377–93. doi: 10.3390/ijms16047377 (PMC4425023; doi:10.3390/ijms16047377)
Supplement: Supplementary file 1 [file ijms-16-07377-s001.pdf]

Supplementary Information

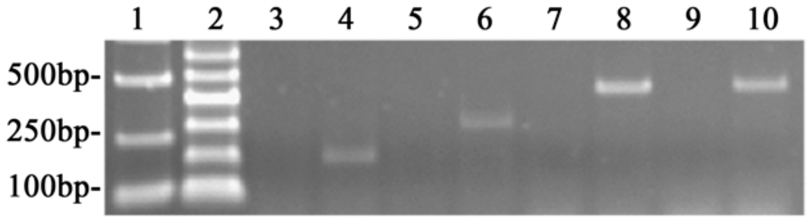

Figure S1. Expression of endogenous and introduced *Gck* and *Gckr* mRNAs in L-02 cells and expression of *Gck* and *Gckr* from transfected plasmids in cells. *Gck* and *Gckr* mRNA levels were examined by PCR with primers that distinguished between the plasmid and endogenous gene-derived mRNAs. **Lane 1**: Marker V; **Lane 2**: PCR marker; **Lane 3**: No *Gck* mRNA (L-02 cells with primers in the *Gck* CDS region); **Lane 4**: *Gck* mRNA (L-02 cells transfected with GCK expressing plasmids with primers in the *Gck* CDS region); **Lane 5**: No *Gck* mRNA (L-02 cells with one primer in the *Gck* CDS region and one in the vector); **Lane 6**: *Gck* mRNA (L-02 cells transfected with GCK expressing plasmids with one primer in the *Gck* CDS region and the other in the vector); **Lane 7**: No *Gckr* mRNA (L-02 cells with primers in the *Gckr* CDS region); **Lane 8**: *Gckr* mRNA (L-02 cells transfected with GCKR expressing plasmids with primers in *Gckr* CDS region); **Lane 9**: No *Gckr* mRNA (L-02 cells with one primer in the *Gckr* CDS region and the other in the vector); **Lane 10**: *Gckr* mRNA (L-02 cells transfected with GCKR expressing plasmids with one primer in the *Gckr* CDS region and the other in the vector).

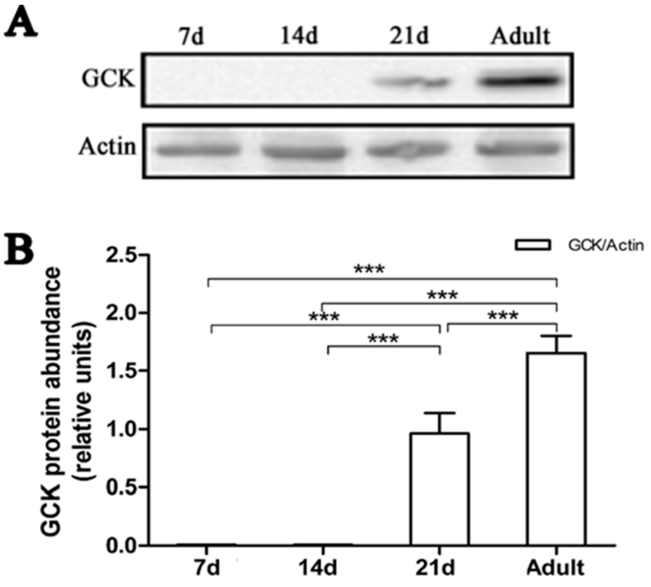

Figure S2. Glucokinase expression levels in the liver of the rat at different ages. (**A**) Representative Western blot for GCK, with β-actin used as a loading control, from liver extracts of rats at 7, 14, and 21-days of age and adults; and (**B**) Quantification of the immunoblots for glucokinase from rats of different ages. All rats were fed ad-lib. Relative units of GCK protein abundance in the Western blots were the abundance of GCK normalized to level of β-actin from the same extract. *** $p < 0.001$. Data presented as means ± S.D. ($n = 4$).

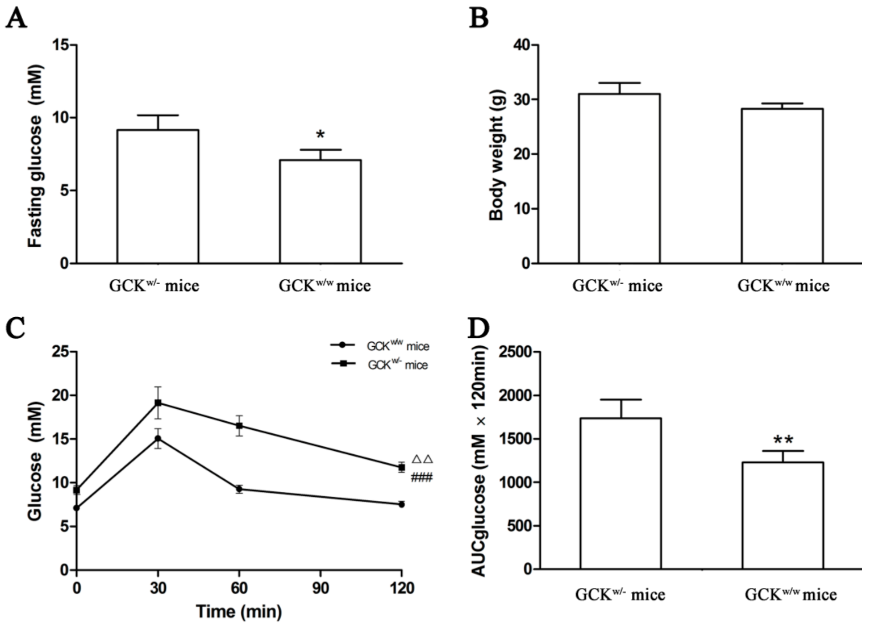

Figure S3. Diabetic character of the liver-specific *Gck* gene knockout mice. (**A**) Fasting blood glucose concentrations; (**B**) Body weights; (**C**) Intraperitoneal glucose tolerance tests; and (**D**) Total AUC calculated from IPGTT tests, are shown for *Gck*$^{w/-}$ and *Gck*$^{w/w}$ mice. Fasting blood glucose was obtained after an 8 h fast, with glucose levels measured at 0, 30, 60 and 120 min after the glucose injection for the IPGTT. * $p < 0.05$, ** $p < 0.01$ *vs*. age-matched *Gck*$^{w/-}$ mice. $^{\Delta\Delta}$ $p < 0.01$ *vs*. age-matched *Gck*$^{w/-}$ mice at 0 min. $^{\#\#\#}$ $p < 0.001$ *vs*. age-matched *Gck*$^{w/w}$ mice at 120 min. Data are presented as means ± S.D. ($n = 4$).

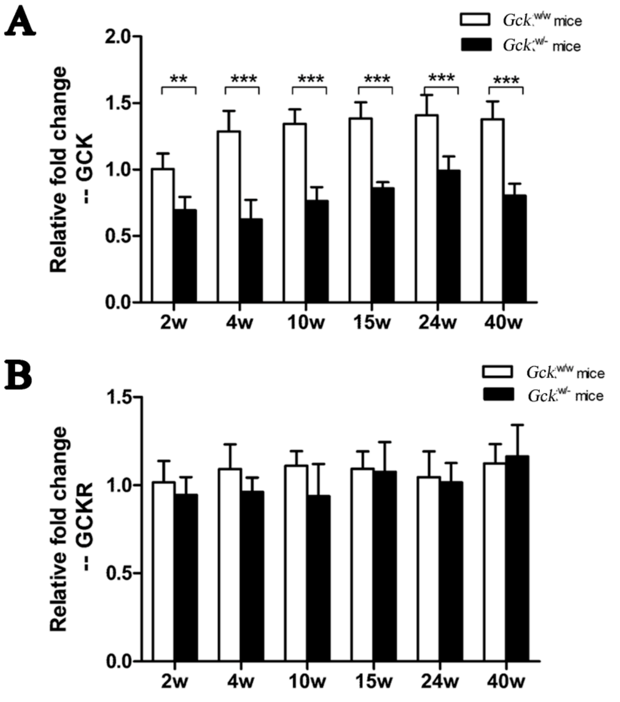

Figure S4. *Gck* and *Gckr* mRNA levels in the liver of *Gck*$^{w/-}$ and *Gck*$^{w/w}$ mice of different ages. (**A**) *Gck* and (**B**) *Gckr* mRNA levels were examined in the livers of wild-type (open bars) and liver-specific *Gck* knockout (solid bars) mice at 2, 4, 10, 15, 24, and 40 weeks of age by real-time PCR. ** $p < 0.01$, *** $p < 0.001$. Data are presented as means ± S.D. ($n = 4$).
